# Data on metal contents (As, Ag, Sr, Sn, Sb, and Mo) in sediments and shells of *Trachycardium lacunosum* in the northern part of the Persian Gulf

**DOI:** 10.1016/j.dib.2016.06.065

**Published:** 2016-07-06

**Authors:** Vahid Noroozi Karbasdehi, Sina Dobaradaran, Iraj Nabipour, Hossein Arfaeinia, Roghayeh Mirahmadi, Mozhgan Keshtkar

**Affiliations:** a Department of Environmental Health Engineering, Faculty of Health, Bushehr University of Medical Sciences, Bushehr, Iran; b The Persian Gulf Marine Biotechnology Research Center, The Persian Gulf Biomedical Sciences Research Institute, Bushehr University of Medical Sciences, Bushehr, Iran; c Systems Environmental Health, Oil, Gas and Energy Research Center, The Persian Gulf Biomedical Sciences Research Institute, Bushehr University of Medical Sciences, Bushehr, Iran; d The Persian Gulf Tropical Medicine Research Center, The Persian Gulf Biomedical Sciences Research Institute, Bushehr University of Medical Sciences, Bushehr, Iran; e Environmental Health Department, School of Public Health, Iran University of Medical Sciences, Tehran, Iran

**Keywords:** Heavy metal, Persian Gulf, Sediment, *Trachycardium lacunosum*

## Abstract

In this data article, by using inductively coupled plasma optical spectrometry (ICP-OES)^1^, we aimed to (1) determine the concentration levels of As, Ag, Sr, Sn, Sb, and Mo in the sediments and the shells of *Trachycardium lacunosum* simultaneously in two separated areas (unpolluted and polluted areas) (2) comparison between the metal contents of sediments in the unpolluted and polluted areas as well as shells. Analysis of data showed that sediment as well as shell samples in polluted area contained significantly higher concentration levels of all measured metals compared with unpolluted area.

**Specifications Table**

**Table:** 

*Subject area*	*Toxicology*
*More specific subject area*	*Heavy metal contents in marine environment*
*Type of data*	*Table and figure*
*How data was acquired*	*ICP*-*OES* (*SPECTRO* (*Germany*)*, Spectro arcos*)
*Data format*	*Raw, analyzed*
*Experimental factors*	*All sediment samples were dried at* 105 °C *for* 24 h, *homogenized, and packed in polyethylene bags and stored at* −20 °C *prior to analysis. The shell samples washed under a jet of tap water to remove algae, sand, clay and other impurities, and then dried at* 105 °C *for* 24 h *and stored at* −20 °C *prior to analysis*
*Experimental features*	*Evaluate the metal contents of As*, *Ag*, *Sr*, *Sn*, *Sb*, *and Mo in sediments and shells of Trachycardium lacunosum in the northern part of the Persian Gulf*
*Data source location*	*Bushehr*, *Asaluyeh bay*, *along the Persian Gulf*, *Iran*
*Data accessibility*	*Data is with this article*

**Value of the data**

• Data can be used as a base-line data for metal concentration levels in marine environments and understanding industrial activities effects on these environments.
• Data shown here can be useful for policy makers, managers, and all related stakeholders, companies, agencies, and institutes working in the fields of environment by imposing proper measures to protect environment.
• Data shown here may serve as benchmarks for other groups working or studying in the field of effluent disposal, pollution control, aquatic ecosystem, toxicology.

## 1 Data

**Fig. 1:**
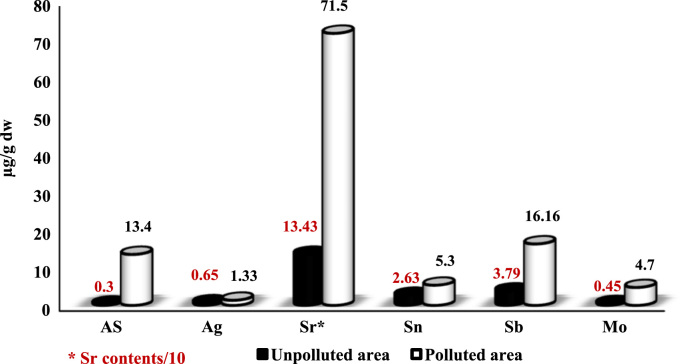
Comparison of metals concentration levels in the sediment samples in polluted and unpolluted areas.

**Fig. 2:**
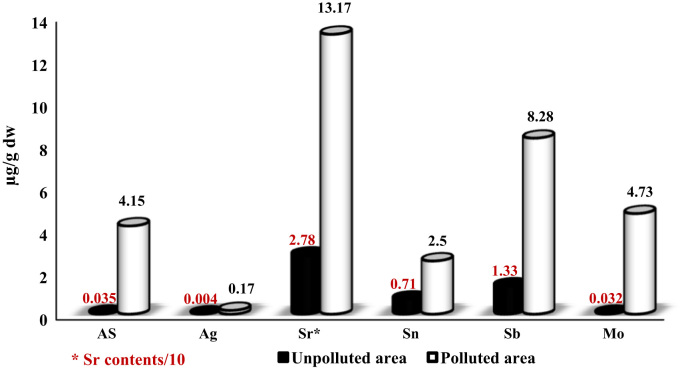
Comparison of metals concentration levels in the shell samples in polluted and unpolluted areas.

In the unpolluted area the concentration levels of As, Ag, Sr, Sn, Sb, and Mo in sediment samples ranged from 0.07–0.81 (Mean: 0.3), 0.4–0.9 (Mean: 0.65), 85–172 (Mean: 134.3), 1.3–3.6 (Mean: 2.63), 2–9.4 (Mean: 3.79) 0.24–0.85 (Mean: 0.45) µg g^−1^ respectively while in the polluted area the concentration levels of As, Ag, Sr, Sn, Sb and Mo ranged from 11.2–16.3 (Mean: 13.4), 1.1–1.7 (Mean: 1.33), 249–1354 (Mean: 715), 4.12–8.32 (Mean: 5.3), 5.9–20.7 (Mean: 16.16), 1.29–9.52 (Mean: 4.7) µg g^−1^ respectively (Table 1 and Fig. 1). In the unpolluted area the concentration levels of As, Ag, Sr, Sn, Sb, and Mo in shell samples ranged from 0.01–0.06 (Mean: 0.035), 0.001–0.008 (Mean 0.004), 21–45 (Mean: 27.8), 0.1–1.6 (Mean: 0.71), 0.5–2.7 (Mean: 1.33), 0.01–0.05 (Mean: 0.03) µg g^−1^ respectively while in the polluted area the concentration levels of As, Ag, Sr, Sn, Sb and Mo ranged from 1.32–6.6 (Mean: 4.15), 011–0.33 (Mean: 0.17), 78–185 (Mean: 131.7), 2.1–3.3 (Mean: 2.5), 3.9–13.8 (Mean: 8.3), 0.7–9.4 (Mean: 4.7) µg g^−1^ respectively (Table 2 and Fig. 2).

## 2 Experimental design, materials and methods

### 2.1 Study area description

**Fig. 3:**
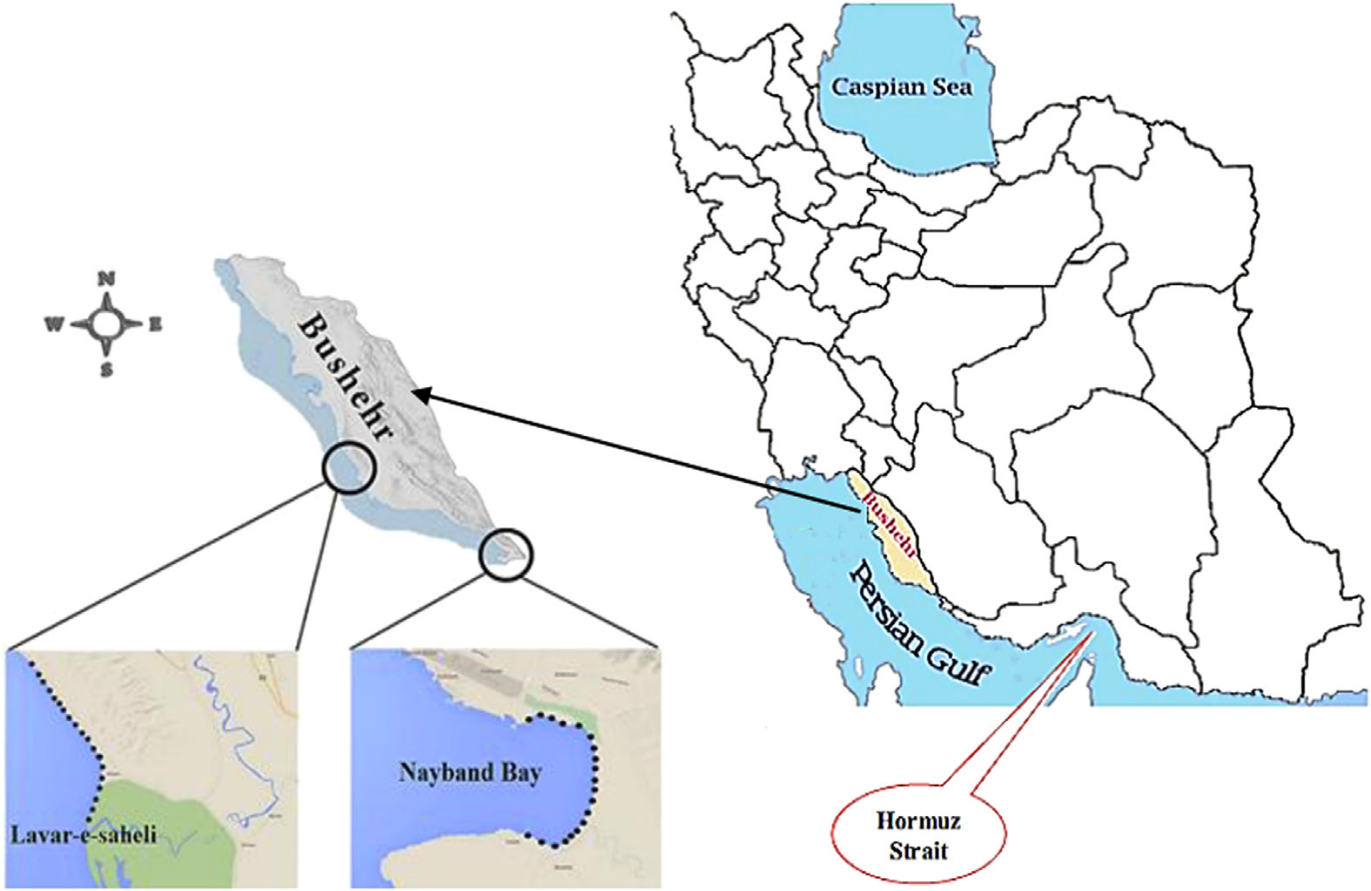
The map and locations of sampling station.

Two different areas were selected in the Asaluyeh as sampling points including polluted area (Nayband Bay) and unpolluted area (Lavar-e-Saheli) (Fig. 3).

### 2.2 Sample collection

Samples from surface sediments (0–10 cm) and shells of *Trachycardium lacunosum* in selected polluted and unpolluted areas were collected. 20 sediment samples and 18 shell samples in polluted area and 19 sediment samples and 13 shell samples in unpolluted area were collected during summer 2013. After transferring the collected sediment samples to the laboratory, the samples were dried at 105 °C for 24 h, homogenized, and packed in polyethylene bags and stored at −20 °C prior to analysis. The shell samples washed under a jet of tap water to remove algae, sand, clay and other impurities, and then dried at 105 °C for 24 h and stored at −20 °C prior to analysis.

### 2.3 Reagents

All the employed oxidants and mineral acids including HNO_3_, H_2_O_2_, HF, HClO_4_ and HCl were of suprapure quality (Merck, Darmstadt, Germany). All glassware and plastic were cleaned by soaking overnight in a 10% (w/v) HNO_3_ solution and then rinsed with deionized water before use. All solutions were prepared by using ultrapure water (18.2 MΩ cm).

### 2.4 Digestion and analytical procedures

The sediment samples (0.5 g) were digested with 2 ml HNO_3_ (65%), 6 ml HCl (37%) in a microwave digestion system for 30 min and then diluted to 25 ml with ultrapure water and stored in polyethylene bottle until analysis. 0.5 g of powdered shell was completely digested in a Teflon cup using a mixture of conc. HNO_3_, HClO_4_ and HF with the ratio 3:2:1 respectively. Acids were slowly added to dried sample and left overnight before further process. Then, the samples were heated at 200 °C then left to cool and filtered. The filtered solution was justified to a volume of 25 ml. Blank digest was also performed in the same way. ICP-OES instrumental method [1], [2], [3] was used to determine the concentration level of metals including As, Ag, Sr, Sn, Sb and Mo (Table 3).

## Figures and Tables

**Table 1:** Concentration levels of metals (µg g^−1^ dw) in sediment samples in polluted and unpolluted areas.

**Area**	**Station**	**AS**	**Ag**	**Sr**	**Sn**	**Sb**	**Mo**
	**1**	0.13	0.6	95	2.3	2.1	0.5
	**2**	0.16	0.7	121	2.1	2	0.35
	**3**	0.20	0.6	99	2.2	3.1	0.24
	**4**	0.20	0.6	145	2.2	4.2	0.36
	**5**	0.25	0.7	152	2.2	3.7	0.41
	**6**	0.33	0.8	136	3.1	2.8	0.61
	**7**	0.52	0.8	137	3.2	6.4	0.74
	**8**	0.70	0.9	128	2.1	7.8	0.71
	**9**	0.81	0.4	85	3.4	9.4	0.39

**Unpolluted area**	**10**	0.79	0.6	154	3.3	8.1	0.42
	**11**	0.46	0.4	126	3.6	2.1	0.54
	**12**	0.10	0.5	126	3.5	2.2	0.57
	**13**	0.14	0.6	146	2.1	2.6	0.85
	**14**	0.07	0.7	137	2.5	2.2	0.24
	**15**	0.17	0.8	152	2.4	4.7	0.36
	**16**	0.14	0.9	163	1.9	2.1	0.35
	**17**	0.21	0.5	172	3.4	2.1	0.24
	**18**	0.14	0.6	132	3.2	2.2	0.24
	**19**	0.13	0.7	146	1.3	2.2	0.41

**Mean±SD**		**0.3±0.24**	**0.65±0.15**	**134.3±22.7**	**2.63±0.67**	**3.79±2.38**	**0.45±0.18**
	**20**	12.5	1.1	325	4.23	5.9	1.5
	**21**	12.3	1.1	452	4.5	19.3	1.65
	**22**	13.5	1.2	489	4.78	19.4	1.75
	**23**	14.2	1.2	1234	5.36	16	1.71
	**24**	11.2	1.3	249	8.23	17.4	3.25
	**25**	11.3	1.1	895	6.32	14.8	2.75
	**26**	12.6	1.4	1124	7.32	16.9	4.53
	**27**	14.3	1.5	1243	5.6	15.3	5.21
	**28**	15.3	1.4	865	4.23	15.3	3.22

**Polluted area**	**29**	11.2	1.6	875	4.56	16.4	6.52
	**30**	13.8	1.7	652	4.12	17	7.21
	**31**	11.6	1.3	452	4.25	18.4	6.32
	**32**	12.6	1.3	365	4.34	20.7	8.21
	**33**	12.5	1.2	263	5.32	16.4	5.32
	**34**	14.6	1.5	965	8.32	16.2	4.38
	**35**	16.2	1.6	1354	7.54	13.6	1.29
	**36**	11.7	1.5	562	4.23	17.8	7.98
	**37**	15.3	1.3	786	4.12	14.8	8.31
	**38**	16.3	1.1	896	4.21	15.3	9.52
	**39**	14.9	1.1	254	4.33	16.2	3.22

**Mean±SD**		**13.4±1.7**	**1.33±0.192**	**715±357.6**	**5.30±1.45**	**16.16±2.99**	**4.7±2.6**

**Table 2:** Concentration levels of metals (µg g^−1^ dw) in shell samples in polluted and unpolluted areas.

**Area**	**Station**	**AS**	**Ag**	**Sr**	**Sn**	**Sb**	**Mo**
	**1**	0.02	0.002	35	0.3	0.9	0.01
	**2**	0.03	0.001	22	0.1	1.2	0.03
	**3**	0.01	0.001	21	0.2	1.1	0.02
	**4**	0.04	0.003	25	0.2	2.2	0.03
	**5**	0.06	0.001	31	1.2	2.7	0.04
**Unpolluted area**	**12**	0.02	0.006	22	1.5	1.3	0.05
	**13**	0.03	0.006	23	0.1	2.2	0.05
	**14**	0.06	0.007	26	0.5	1.2	0.03
	**15**	0.04	0.008	35	0.4	0.7	0.02
	**16**	0.02	0.006	45	1.6	0.5	0.04
	**17**	0.03	0.001	22	1.4	1.3	0.03
	**18**	0.04	0.002	26	0.2	1.2	0.04
	**19**	0.05	0.003	28	1.5	0.8	0.03
**Mean±SD**		**0.035±0.016**	**0.004±0.003**	**27.8±7.04**	**0.71±0.62**	**1.33±0.65**	**0.032±0.012**
	**20**	2.5	0.12	150	2.6	3.9	0.7
	**22**	3.3	0.23	185	3.1	7.4	1.7
	**23**	3.2	0.21	97	2.4	5.3	1.8
	**24**	4.2	0.33	144	3.2	12.4	3.3
	**25**	1.3	0.22	125	2.3	11.8	2.6
	**26**	2.9	0.21	125	3.3	10.9	4.4
	**27**	4.7	0.11	85	2.6	4.3	5.6
	**28**	5.3	0.12	162	2.2	6.3	3.4
**Polluted area**	**29**	1.6	0.13	168	2.5	9.4	6.3
	**31**	2.6	0.15	139	2.2	9.4	6.9
	**32**	6.6	0.25	145	2.3	6.7	8.3
	**33**	4.5	0.26	99	2.3	6.4	5.7
	**34**	6.6	0.11	78	2.3	6.2	4.6
	**35**	6.2	0.12	135	3.2	5.6	1.3
	**36**	5.7	0.12	85	2.2	5.8	7.2
	**37**	5.3	0.13	185	2.1	13.8	8.3
	**38**	4.3	0.14	136	2.2	11.3	9.4
	**39**	3.9	0.15	128	2.3	12.2	3.6
**Mean±SD**		**4.15±1.6**	**0.17±0.06**	**131.7±32.8**	**2.5±0.4**	**8.28±3.12**	**4.73±2.63**

**Table 3:** ICP-OES instrumental operating details.

**Parameters**	
Company, model	SPECTRO (Germany), Spectro arcos
RF generator power (W)	1400
Frequency of RF generator (MHz)	27.12 MHz
Type of detector	Charge coupled devices (CCD)
Torch type	Flared-end EOP torch 2.5 mm
Plasma, auxiliary, and nebulizer gas	High purity (99.99%) argon
Plasma gas flow rate (L min^−1^)	14.5
Auxiliary gas flow rate (L min^−1^)	0.9
Nebulizer gas flow rate (L min^−1^)	0.85
Sample uptake time (s)	240 total
Delay time of (s)	–
Rinse time of (s)	45
Initial stabilization time (s)	Preflush: 45
Time between replicate analysis (s)	–
Measurement replicate	3
Pump rate	30 RPM
Element (*λ* nm^−1^)	Ag 328.068; As 189.042; Sr 346.446
	Sn 189.991; Sb 206.833; Mo 204.598
