# Supplementary material for: Data on metal contents (As, Ag, Sr, Sn, Sb, and Mo) in sediments and shells of Trachycardium lacunosum in the northern part of the Persian Gulf
Source: Data Brief. 2016 Jul 6;8:966–71. doi: 10.1016/j.dib.2016.06.065 (PMC4961305; doi:10.1016/j.dib.2016.06.065)
Supplement: Supplementary file 1 — Supplementary material [file mmc1.docx]

Bushehr University of Medical Sciences

**Dear Editor of Data in Brief**

The research reported in this manuscript has been funded through the Bushehr University of Medical Sciences. This manuscript has not been previously published and is not under consideration in the same or substantially similar form in any other peer-reviewed media. All authors listed have contributed sufficiently to the project to be included as authors. All authors have read the manuscript and have agreed to submit it in its current form for consideration for publication in the Journal.

**The authors declare that they have no conflict of interest, financial or other.**

Sincerely Yours,

Sina Dobaradaran, Ph.D.

Associate Professor
Department of Environmental Health Engineering, Faculty of Health &
The Persian Gulf Marine Biotechnology Research Center, Bushehr
University of Medical Sciences, Bushehr, Iran.
Mobile: 0098-917-373-1242
